# The Association of Home Environment and Caregiver Factors With Neurocognitive Function in Pre-school- and School-Aged Perinatally Acquired HIV-Positive Children on cART in South Africa

**DOI:** 10.3389/fped.2019.00077

**Published:** 2019-03-26

**Authors:** Antonio G. Lentoor

**Affiliations:** Department of Clinical Psychology, School of Medicine, Sefako Makgatho Health Sciences University, Pretoria, South Africa

**Keywords:** HIV, children, neurocognitive function, home environment, cART, biological and non-biological (extended relatives) caregivers

## Abstract

**Background:** Children with perinatally acquired HIV in low resource settings are at risk for neurocognitive impairments not only due to the direct effects of HIV on the brain and *in utero* ART exposure but also due to factors associated with their environment. Thus, the aim of this study was to examine the association between home environment and caregiver factors and the neurocognitive function of pre-school- and-school-aged HIV-positive South African children from low resource rural communities.

**Materials and Methods:** The Wechsler Preschool and Primary Scale of Intelligence-III was administered to assess the neurocognitive functioning of 152 purposively sampled perinatally acquired HIV-positive children on cART, aged 3 years to 7 years 6 months (mean age 63.13 months). The primary caregivers (*n* = 152) completed the Home Screening Questionnaire to assess the quality of the home-environment of the children.

**Results :**The results showed that unfavorable environment, caregiver type, and quality of stimulation in the home were negatively associated with the neurocognitive development of perinatally HIV-positive children on cART. Most of the HIV-positive children (*n* = 95) were under the care of an extended relative. Older HIV-positive boys, reared by biological mothers, who also lived in suboptimal and poor quality home-environments had poorer neurocognitive function when compared to HIV-positive children reared by non-biological (extended relatives) caregivers, [*F*_(2,149)_ = 14.42, *p* < 0.001].

**Conclusion:** The child's early home environment is associated with general neurocognitive development, which highlights the need for early psychosocial interventions that can promote better cognitive outcomes among children living with HIV.

## Introduction

With the global availability of combination antiretroviral therapy (cARTs) to perinatally acquired HIV-positive children, we witnessed a decline in premature death, and more children living with HIV now have a greater chance of growing into school-age, adolescence, and even adulthood (1, 2). Not only did cARTs have a definitive effect on the progression of the disease but they also significantly decreased incidences of early severe central nervous system disease (3).

As access to HIV treatment has expanded throughout South Africa and Africa (4), not only has treatment prolonged survival amongst children but it also has promoted growth, development and an overall improvement in the quality of life of children living with HIV. However, despite the noticeable improvement in the survival rate of HIV-positive children with cART, an increasing number of pre-school- and school-age HIV-positive children are being identified with neurocognitive impairments (5, 6) Neurocognitive impairments have been observed as early as infancy and continue into school-age years, and can manifest as global delays. Problems with executive functioning, speed and information processing, attention and working memory, language and sensory perception, and psychomotor skills are observed in children living with HIV (7).

A systematic review of published work in Africa showed that neurodevelopmental delays in perinatally HIV-positive children were related to socioenvironmental risk factors and not entirely due to the HIV infection (8). Psychosocial factors such as suboptimal home environment (9), inadequate social support, caregiver unemployment (10) and maternal depression (11) are all factors that have been shown to influence neurocognitive function of children in addition to HIV. Other factors associated with neurocognitive deficits and developmental delays in HIV-positive children were maternal health, maternal education, and age of primary caregiver (12).

There is a dearth of knowledge on the factors influencing the neurocognitive functioning of perinatally acquired HIV-positive children in South Africa (13). It is important to ascertain the factors, besides HIV, that may be associated with the neurocognitive development of HIV-positive children living in low resource settings, especially as they are surviving beyond infancy. This biopsychosocial understanding of pediatric health is pivotal, especially since children with neurocognitive deficits have been shown to have trouble with learning and concentration, which inadvertently affects their schooling and productivity and has implications for overall educational achievements and psychosocial functioning (11). Neurocognitive function has also been found to be associated with treatment adherence (14) and therefore this understanding is crucial amongst HIV-positive children, as they grow older and eventually transition into taking control over their own health (15). Without understanding the associated psychosocial factors that continue to remain a challenge for children living with HIV in South Africa and other low resource settings, we will fail to tailor adequate interventions necessary for this population. Therefore, the purpose of this study was to describe the association between home environment, caregiver factors, and the neurocognitive function in a group of perinatally acquired HIV-positive pre-school- and school-age children from a low resource rural South African community.

## Materials and Methods

### Study Location

A cross-sectional study was conducted among perinatally acquired HIV-positive children and their caregivers at a local academic hospital within the Buffalo City Metropolitan Municipality, Eastern Cape, South Africa. The Eastern Cape is one of the poorest provinces in South Africa, with a high level of under development and an unemployment rate of about 24.3% (16). Children below the age of 18 years have health care provided through the Department of Pediatrics at the health facility. The facility provided both inpatient pediatric services and pediatric services via the outpatient HIV Clinic for HIV-positive children from across rural and urban Eastern Cape.

### Study Participants

A sample of 152 child/caregiver dyads were purposively recruited in a consecutive fashion as they attended the outpatient's clinic. Both, biological and non-biological caregivers of children with perinatally acquired HIV and treated on combination anti-retroviral dugs (cARTs) who attended the Pediatric Outpatient HIV Clinic was invited to participate in the study. The sample of perinatally HIV-positive children at pre-school- and school-age ranging from 31.38 to 92.78 months (*M* = 63.13) was accessing outpatient care at the Pediatric Clinic on a monthly basis and was comprised of both girls (*n* = 87) and boys (*n* = 65). Caregivers whose children were HIV-positive and were in the age range of 31.38–92.78 months (*M* = 63.13), were included in the study.

Inclusion criteria

1. Confirmed HIV-positive diagnosis and on cART
2. Age ½−7 years
3. Informed consent by the primary caregiver

Exclusion criteria

1. Acute illness and/or hospitalization at the time of neurocognitive test administration
2. Chronic neurological disorders such as epilepsy, cerebral palsy, head injury, meningitis and congenital infections
3. Attendance of specialized schooling

## Measures

### Socio-Demographic Data

Socio-demographic information, as informed through a literature search of commonly associated socio-economic factors that influence neurodevelopment outcomes in children from similar social context, was collected. This included information regarding the child's age, sex and ethnicity, the child's relationship to the primary caregiver, as well as the highest level of education, employment status, family size, income, social grant uptake, and the marital status of primary caregiver. The research also collected information on housing, access to water, sanitation, electricity, appliances (e.g., fridge, washing machine, etc.).

### Home Screening Questionnaire (HSQ)

The Home Screening Questionnaire (HSQ), a self-report questionnaire based on the Home Measurement of the Environment (HOME) Inventory was used to assess the overall quality of the home environment in which the children were reared (17, 18). The 34-item questionnaire taps into home environmental characteristics such as parental involvement, family activities, organization, discipline, and resources available for physical stimulation in the home. The total score is the sum of the questionnaire. A home environment characterized by a lack of stimulating qualities would likely have few written and reading materials or children's toys available, and adult caregivers in these homes are likely to engage in very limited stimulating activities with the child, which will be reflected by a score of 41 or below. The Home Screening Questionnaire has been widely used as a tool that identifies home environments likely to be suboptimal for child development in South Africa (19, 20) and other developing countries (21). The HSQ demonstrated an acceptable reliability in this study sample of HIV-positive children, with a Cronbach's internal consistency reliability coefficient of 0.61.

### Wechsler Preschool and Primary Scale of Intelligence-III (WPPSI-III)

The Wechsler Preschool and Primary Scale of Intelligence-III (WPPSI-III) test is an assessment tool that was developed for children of ages of 2½ years and 7 years and 3 months (22). It assesses cognitive functioning in pre-school and primary-school-age children. The WPPSI-III provides subtests and composite scores that represent functioning in a wide range of neurocognitive domains such as Verbal IQ (VIQ) and Performance IQ (PIQ), as well as providing a composite score that represents a child's general intellectual ability (i.e., Full Scale Intellectual Quotient (FSIQ). The WPPSI-III as a measure of neurocognitive function has been applied to HIV research in children from low-income backgrounds and is representative of the larger population of children living with HIV (23). It should be noted that Quotient and Composite scores have a mean of 100 and a standard deviation of 15, while subtest-scaled scores have a mean of 10 and a standard deviation of three. Neurocognitive impairment was defined as a score of −2SD below the mean (100) for the WPPSI-III (24). In the absence of South African normative data, standardization was based on US disadvantage norms. Average internal consistency reliability for the full scale IQ is 0.87 and adequate concurrent validity has been established.

### Procedures and Data Collection

Children with perinatally acquired HIV and their primary caregiver attending the Pediatric Outpatient HIV Clinic (POHC) who satisfied the inclusion criteria were recruited to participate in the study. Primary caregivers were approached in the POHC after the Head of the Pediatric Department granted formal access. All primary caregivers provided written informed consent for themselves and their children. The research was explained to the caregivers in English by a clinical psychologist, and in isiXhosa by a clinical social worker, depending on the caregiver's language preference; both had prior experience of working with HIV/AIDS-infected children and parents. Information on the children's health was directly obtained from their clinical folder and confirmed by the caregivers, while the sociodemographic data and home environment history of the children was obtained from the caregivers. The caregivers completed the Home Screening Questionnaire at home in an interview format administered by the clinical social worker who had relevant experience in administering the Home Screening Questionnaire. All the children participating in the study completed the WPPSI-III as a measure of neurocognitive functioning in pre-school and school age children. With the children, the registered clinical psychologist with relevant training and experience in child neuropsychological assessment administered the WPPSI-III during their routine check-up at the clinic in a private consulting room.

### Statistical Analysis

The statistical package IBM SPSS (21.0) (25) was used to analyse the data. The Shapiro-Wilk test was conducted to explore the data and to observe the assumptions of normality in the data, while the Levene's test was used to assess assumptions of equality or homogeneity of variance in the variables. Pearson's (*r*) was performed to determine the associations between the neurocognitive functioning and home environment of the HIV positive children. Group comparisons between biological and non-biological caregivers, age of caregivers and children, and gender of children in relation to the children's neurocognitive function and quality of home environment were assessed using the one- way analysis of variance test (ANOVA) and independent sample *t*-test. All tests conducted were two-tailed and held statistical significance at *p* < 0.05.

## Results

### Sociodemographic Characteristics

The sociodemographic characteristics of the participants are presented in Table 1. All the children in the sample had perinatally acquired HIV (*n* = 152) and were treated with antiretroviral therapy. Extended family members, such as the grandmother and aunts, who were the main caregivers of the children, were in the majority (*n* = 96, 63%), while only 37% (*n* = 56) were the biological mothers of the children. All the biological mothers were living with HIV, which was confirmed during the consenting process at baseline. Caregivers were generally middle-aged (*M* = 45 years, range = 18–79 years, *p* < 0.01) women with the majority (65%) having obtained at least a high school education (*p* = 0.01). Only 7.2% (*n* = 11) of the caregivers had casual employment, while more than 90% (*n* = 141) relied on a child support or old age government grant as their sole source of income (*p* < 0.01). Caregiver age, education, and employment were lower for biological caregivers than non-biological group (*p* < 0.01).

**Table 1:** Sociodemographic characteristics of HIV-positive children and caregiver dyads (N = 152) in the study.

**Characteristics**	**Non-biological caregivers, *n* = 95**	**Biological caregivers, *n* = 57**	***p*-value**
**AGE OF CHILD (IN MONTHS)**			
Mean ±*SD*	64.58 ±16.12	60.73 ±18.09	0.51
**SEX**			
Male	44 (46.3)	21 (36.8)	0.25
Female	51 (53.7)	36 (63.2)	
**CHILD'S LEVEL OF EDUCATION**			
N/A	26 (27.4)	20 (35.1)	0.53
Crèche	7 (7.4)	6 (10.5)	
Grade R	33 (34.7)	13 (22.8)	
Primary	29 (30.6)	18 (31.6)	
**CAREGIVER'S AGE**			
18 years to 35 years	16 (16.8)	37 (64.9)	0.000^**^
36 years and above	79 (83.2)	20 (35.1)	
**CAREGIVER LEVEL OF EDUCATION**			
None	2 (2.1)	3 (5.3)	0.01^*^
Primary	34 (35.8)	11 (19.3)	
Standard 6–8	32 (33.7)	15 (26.3)	
Standard 9–10	26 (27.4)	26 (45.6)	
Tertiary	1 (1.1)	2 (3.5)	
**SES INDICATOR(S)**			
Casual Employment	6 (6.3)	5 (4.8)	0.001^**^
Grant (Disability/Child support/Old age pension)	89 (93.7)	52 (91.3)	

Significance level

* p < 0.05;

** *p < 0.01. SD, standard deviation; SES, socioeconomic status. Level of education: primary schooling (7 years to 11 years), secondary schooling (12 years to 16 years), tertiary (post school education, include college and university)*.

### Neurocognitive Functioning Characteristics of HIV-Positive Children

**Figure 1:**
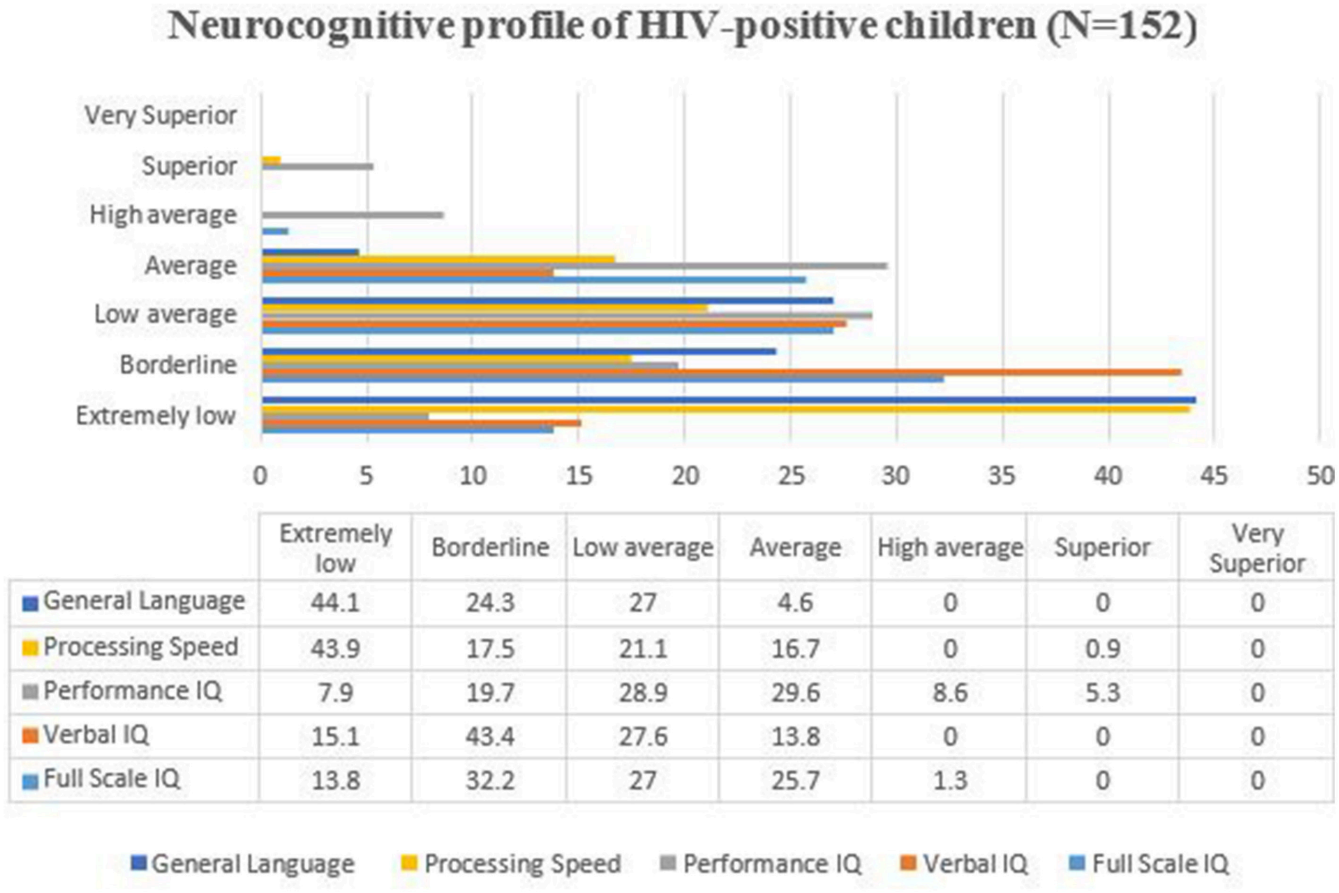
Neurocognitive profile of HIV-positive children in the study. The bars and figures represented the percentages of children in the different domain of IQ. IQ, Intellectual Quotient.

The overall mean scores on the WPPSI-III for the HIV-positive children in this study showed that the children scored consistently low (81.47 ± 12.81). More than half (73%) the sample of children tested using the WPPSI-III fell below the average (100 ± 15), with only 1.3% of the overall sample performing in the high average range (IQ: 110–119) (Figure 1). When matched to the VIQ (13.8%), more children scored within the normative range on PIQ (29.6%) (Figure 1).

### Quality of Home Environment of HIV-Positive Children

The results indicate that all of the children in the study lived in unfavorable home environments (*n* = 152, 100%) based on the HSQ scores (14.69 ± 3.22). The mean score for HIV-positive children living with a biological caregiver was 17.47 ± 5.26, which was significantly (*p* < 0.01) lower than the mean score of 20.27 (± 6.11) for the HIV-positive children living with a non-biological caregiver, as shown in Table 2.

**Table 2:** Independent *t*-test on the sample.

**Variables/dimensions**	**Non-biological caregivers**		**Biological caregivers**		***t***
	**Mean**	***SD***	**Mean**	***SD***	
FSIQ	81.78	13.42	80.96	11.82	0.38
VIQ	77.38	12.26	78.12	9.38	0.39
PIQ	90.39	17.68	88.28	15.79	0.74
HSQ	20.27	6.11	17.47	5.26	2.88^**^
**Variables/dimensions**	**Younger caregiver (18-35 years)**		**Older caregiver (36 and above)**		***t***
	**Mean**	***SD***	**Mean**	***SD***	
FSIQ	80.62	12.41	81.93	13.06	0.59
VIQ	77.38	10.77	77.81	11.54	0.22
PIQ	89.51	15.79	89.65	17.66	0.05
HSQ	19.30	5.80	19.18	6.05	0.12
**Variables/dimensions**	**Boys**		**Girls**		***t***
	**Mean**	***SD***	**Mean**	***SD***	
FSIQ	78.98	12.91	83.33	12.48	2.09^*^
VIQ	76.51	10.49	78.52	11.75	1.09
PIQ	86.08	15.61	92.23	17.56	2.24
HSQ	18.74	6.18	19.59	5.77	0.87

Significance level

* p < 0.05;

** *p < 0.01. FSIQ, Full Scale Intellectual Quotient; VIQ, Verbal Intellectual Quotient; PIQ, Performance Intellectual Quotient; HSQ, Home Screening Questionnaire; SD, standard deviation*.

### Relationship Between Home Environment and Neurocognitive Function

No statistical significant relationship was established between global neurocognitive functioning [*t*_(151)_ = 0.38; *p* > 0.05] and the quality of home environment in the sample of HIV-positive children (Table 2). On subgroup analysis, the results interestingly suggest that the amount of stimulation a child is exposed to in the home environment, as measured on the HSQ, significantly influences the sub-neurocognitive domain of verbal neurocognitive functioning (VIQ) as indicated in their negative association (*r* = −0.18, *p* < 0.05). Family structure, including living with a biological caregiver, lack of resources in home for stimulation, and lower education of caregiver were associated with higher risk for poorer neurocognitive function. Verbal neurocognitive functioning correlated significantly negatively with both lack of stimulation in the form of reading material availability in the home (*r* = −0.24, *p* < 0.05). Overall poor quality of home environment stimulation was found (HSQ) (*r* = −0.21, *p* < 0.05) for HIV-positive children, especially for those children were reared by a biological caregivers (17.47 ± 5.26) compared to non-biological caregivers (20.27 ± 6.11) [*t*_(150)_ = 2.88, *p* < 0.01 two-tailed].

Among the children, age, gender, and living with a biological caregiver were found to be significantly associated with poorer neurocognitive outcomes. The results show that older boys were worse off than older and younger age girls in this study; with boys scoring lower (78.98 ± 12.91) than girls (83.33 ± 12.48) on the neurocognitive functioning scale, [*F*_(2,149)_ = 14.42, *p* < 0.001] (Table 3), especially if they were also living in a suboptimal home-environment with a biological caregiver compared to living with a non-biological caregiver (*p* < 0.01).

**Table 3:** One-way ANOVA results for age group differences among HIV+ children and the key variables in the study.

**Source of variance**	**Mean squares**	***df***	***F* values**	***P***
FSIQ	133.97	2	0.82	0.445
VIQ	1546.97	2	14.42	0.000^***^
PIQ	736.733	2	2.61	0.077
HSQ	516.47	2	17.89	0.000^***^

Significance level

*** *p < 0.01. FSIQ, Full Scale Intellectual Quotient; VIQ, Verbal Intellectual Quotient; PIQ, Performance Intellectual Quotient; HSQ, Home Screening Questionnaire; df, degree of freedom*.

## Discussion

This is the first study to assess the association between aspects of home environment, and caregiver factors with neurocognitive functioning of a large cohort of perinatally HIV-positive pre-school- and school-age children in South Africa. The study demonstrated that pre-school- and school-age perinatally HIV-positive children significantly underperformed on the measure of neurocognitive function (81.47 ± 12.81). This finding concurs with previous studies, which showed that HIV-positive children are at risk for neurocognitive impairment similar to that of HIV-positive pre-school- and school-age children from resource rich settings (26) and resource constrained settings (27). Similarly, to previous studies (28), the children in this sample were on cART and were considered clinically stable as per inclusion criteria. A number of studies have reported that while HAART has shown to arrest the progression of HIV-associated encephalopathy (29) reports of ongoing neurocognitive decline persist due to early *in-utero* HIV infection (30). Evidence suggest, where early infection occurs, antiretroviral drugs may not completely be able to reverse the associated effects of HIV on the brain (26). However, perhaps a plausible alternative explanation could be found in the effects of various aspects of the environment on the children's neurocognitive development (31). Several studies suggested that antiretroviral drugs, in the context of an impoverished environment (32), is not completely protective and reparative. Comparable support for the impact of home environment in a low resource setting was found in the study conducted in Zimbabwe by Kandawasvika and colleagues who compared HIV-positive children on cART with HIV-exposed but uninfected with HIV children from similar home environments, and found a higher prevalence of neurocognitive impairment (18%) in the uninfected compared to HIV-infected group (16%) (33). This finding suggests that it is important to consider the environment given that only a 2 point difference (18 vs. 16%) was observed among the sample of Zimbabwean children. The study showed that when the environment was held constant, the performances on neurocognitive function amongst the two groups were close to similar (only 2 point difference), despite the fact that the HIV-positive children received antiretroviral treatment. It suggests that children, irrespective of HIV status, who happen to also live in suboptimal home environments, are at risk for neurocognitive deficits.

Poorer cognitive functioning among the sample of HIV-positive children was associated with caregiver type, limited cognitively stimulation material and poor quality of stimulation experience. Those children who were raise by a biological caregiver showed poorer cognitive and emotional stimulation in the home (*p* < 0.01) and had fewer toys, and materials necessary for cognitive stimulation. Stimulating materials and experiences has been found to mediate the relationship between poverty, and cognitive outcomes (i.e., intellectual and academic achievement) from infancy to adolescence (34). Biological caregivers of HIV-infected children have shown to face numerous daily stressors associated with being a single parent, unemployed, and HIV-positive, that place their children at greater risk for poor neurodevelopment (35), which may result in less stimulating and supportive home environments necessary for learning opportunity that can promote cognitive growth and resilience in their HIV-positive children (36).

Of importance, the role of caregivers (extended family) other than biological parents living with HIV (37) to provide a protective net for their children, especially if they are living in resource-constrained environments, is partially highlighted. The role of the extended family as an important form of social protection resource, especially for the care of orphans, as it is argued to mitigate the effects of extreme deprivation and vulnerability associated with both poverty and HIV/AIDS (38, 39). However, with the increasing burden of HIV and poverty in resource-constrained settings comes an increase in the burden of care, thus adding strain on already limited psychosocial and household resources available to these non-biological (extended) caregivers. Consequently, any adverse influence on the quality of care of the children stands to compromise any opportunity for the extended caregivers to promote resilience or to provide a safety net amongst an already vulnerable group of children. In accordance with social ecology theory, the family and proximal context remains most influential part of the child's developmental outcomes, in both the context of, and in the absence of HIV (40).

The findings from this study should be interpreted cautiously as it is not without limitations. In particular, the findings are based on a cross-sectional study and preclude drawing causal explanation of any associations. While the study aimed to obtain a sample representative of the black African perinatally HIV-infected pediatric population, researchers should be cautious about generalizing the finding beyond the parameters set by the sample characteristics. The neurocognitive measure also posed an additional limitation, as there was no normative data from South African populations. As a result, the findings needs to be cautiously interpreted since the measure used to assess the children in the study was not validated for the local context. Longitudinal studies are recommended that can standardize these tools for culturally and linguistically diverse resource-constraint settings and investigate potential causal interaction between environmental and biomedical markers (e.g., CD-4 count, viral load, etc.) on child cognitive development. The study findings presented here reinforce a need for a multimodal intervention approach in dealing with HIV given that an impoverished environment is associated with poor neurodevelopment outcome in children living with HIV. At a proximal level, healthcare intervention should incorporate psychosocial interventions that target caregiver-child relationships and strengthening caregivers' abilities to enable them to work toward building resilient capacity in their HIV-positive children. Home-based interventions that include home visits and offering parenting skills training have shown to be promising in resource-constrained settings and may circumvent environmental risk factors such as poverty. More importantly, in addition to the medical care provided for South African HIV-infected children, it is recommended that neuropsychological and psychosocial assessments be incorporated into their overall standard of care. This will not only ensure early detections of neurocognitive and socioemotional problems associated with both the HIV and the socio-environment, but will also result in prompt or timeous psychosocial interventions that stand to improve on the overall quality of life of this population. At a structural level, existing poverty alleviation, supplementation and nutritional programmes should be strengthened to contribute to an enriching home environment, which will improve the long-term outcomes of HIV-positive children in resource-constrained settings.

## Conclusions

The findings from this study concur with previous studies that have argued that factors associated with a child's home environment, such as early quality of child's environment, caregiver relationship as well as child factors are associated with the neurocognitive development of HIV-positive children.

## Ethics Statement

Ethical approval was given by both the University of KwaZulu Natal's Biomedical Research Ethics Committee (Protocol Number: BE252/11) and the Ethics Committee at the Hospital. Each caregiver was informed about the purpose of the study, and it was emphasized that their participation or otherwise would not affect their child's treatment or quality of care at the health facility. Caregivers gave written informed consent for themselves and the children to participate in the study. All other ethical principles (informed consent, anonymity, confidentiality, and voluntary participation) were completely followed. Caregivers were informed of the availability of counseling and social-welfare services should the need for these arise.

## Author Contributions

The author confirms being the sole contributor of this work and has approved it for publication.

### Conflict of Interest Statement

The author declares that the research was conducted in the absence of any commercial or financial relationships that could be construed as a potential conflict of interest.
